# Virus-Like Particle Vaccination Protects Nonhuman Primates from Lethal Aerosol Exposure with Marburgvirus (VLP Vaccination Protects Macaques against Aerosol Challenges)

**DOI:** 10.3390/v8040094

**Published:** 2016-04-08

**Authors:** John M. Dye, Kelly L. Warfield, Jay B. Wells, Robert C. Unfer, Sergey Shulenin, Hong Vu, Donald K. Nichols, M. Javad Aman, Sina Bavari

**Affiliations:** 1 United States Army Medical Research Institute of Infectious Diseases, Fort Detrick, MD 21702, USA; jay.b.wells.ctr@mail.mil (J.B.W.); donald.k.nichols.civ@mail.mil (D.K.N.); sina.bavari.civ@mail.mil (S.B.); 2 Integrated Biotherapeutics, Inc., Gaithersburg, MD 20878, USA; Robert.unfer@nih.gov (R.C.U.); sergey@integratedbiotherapeutics.com (S.S.); hong@integratedbiotherapeutics.com (H.V.); javad@integratedbiotherapeutics.com (M.J.A.)

**Keywords:** Marburg virus, virus-like particle, vaccine, macaque, aerosol, adjuvant

## Abstract

Marburg virus (MARV) was the first filovirus to be identified following an outbreak of viral hemorrhagic fever disease in Marburg, Germany in 1967. Due to several factors inherent to filoviruses, they are considered a potential bioweapon that could be disseminated via an aerosol route. Previous studies demonstrated that MARV virus-like particles (VLPs) containing the glycoprotein (GP), matrix protein VP40 and nucleoprotein (NP) generated using a baculovirus/insect cell expression system could protect macaques from subcutaneous (SQ) challenge with multiple species of marburgviruses. In the current study, the protective efficacy of the MARV VLPs in conjunction with two different adjuvants: QS-21, a saponin derivative, and poly I:C against homologous aerosol challenge was assessed in cynomolgus macaques. Antibody responses against the GP antigen were equivalent in all groups receiving MARV VLPs irrespective of the adjuvant; adjuvant only-vaccinated macaques did not demonstrate appreciable antibody responses. All macaques were subsequently challenged with lethal doses of MARV via aerosol or SQ as a positive control. All MARV VLP-vaccinated macaques survived either aerosol or SQ challenge while animals administered adjuvant only exhibited clinical signs and lesions consistent with MARV disease and were euthanized after meeting the predetermined criteria. Therefore, MARV VLPs induce IgG antibodies recognizing MARV GP and VP40 and protect cynomolgus macaques from an otherwise lethal aerosol exposure with MARV.

## 1. Introduction

The Filoviridae family consists of two genera of viruses denoted Ebolavirus and Marburgvirus, which are non-segmented, negative-strand RNA viruses. Filovirus infection of nonhuman primates and humans causes a highly lethal hemorrhagic fever disease with case mortality rates of 30%–90% [1]. Marburgviruses were named for the location of the first recognized filovirus outbreak that took place in Marburg, Germany in 1967. The outbreak originated in imported, infected monkeys from Uganda with diseases occurring primarily in vaccine production workers processing tissues [2,3]. The Marburgvirus genus consists of two species, Marburg Marburgvirus (MARV) and Ravn Marburgvirus (RAVV) [4]. Since the original outbreak, less than 50 total cases of MARV or RAVV were reported until a large outbreak having ~50% case mortality occurred during 1998–2000 in the Democratic Republic of Congo followed by a second outbreak in 2005 in Northern Angola where the case mortality rates were 80%–90% [5,6,7]. More recently, there have been outbreaks in 2012 and 2014 both originating in the Kabale and Ibanda regions of Uganda [8].

Numerous vaccine platforms have been assessed for efficacy against lethal Marburgvirus exposure over the last 30–40 years [9]. Multiple species of rodents and nonhuman primates have been used as animal models of Marburg hemorrhagic fever in humans; however, rhesus and cynomolgus macaques exhibit disease progression similar to humans and, therefore, are the most utilized models for assessing vaccine efficacy [10,11]. Classical vaccine approaches, such as inactivated viruses, were attempted in early endeavors with mixed success in nonhuman primate models [12,13], while more recent novel approaches using innovative molecular biology techniques have shown more promise [14]. Replication-deficient virus-vectored candidates such as the Venezuelan equine encephalitis virus replicon particle (VRP) and adenoviruses encoding the protective glycoprotein (GP) antigen with or without the viral nucleoprotein were amongst the first vaccine platforms to show success [15,16,17]. Vaccination with DNA alone can protect against lethal MARV infection in macaques and DNA prime with a virus vectored vaccine boost seems to provide stronger immunological responses than DNA alone [18]. GP-expressing replication-competent virus vectors such as vesicular stomatitis virus, human parainfluenza virus type 3, and rabies virus have also demonstrated protection against subsequent filovirus infections in macaques [19,20,21,22]. Recently, virus-like particles (VLPs) were also shown to protect vaccinated macaques against parenteral ebolaviruses and MARV challenge [23,24].

VLPs mimic the particle structure of the authentic native viruses, present the protective viral antigens to the immune system with the correct conformation, but lack a viral genome; thus, these properties engender VLPs to be safe, economical, potent vaccine candidates. In the past several years, VLPs have risen in the vaccine field as a promising vaccine platform for a diverse set of viral pathogens including both nonenveloped and enveloped viruses [25,26,27,28,29]. Encouragingly, a number of VLP-based vaccines are licensed for human use including Engerix^®^ and Recombivax HB^®^ hepatitis B vaccines as well as Cervarix^®^ and Gardasil^®^ human papillomavirus-like particle-based vaccines for prevention of cervical cancer [30].

Expression of the filovirus matrix protein, viral protein 40 (VP40), is sufficient to drive budding of filovirus-like particles [31]. However, addition of GP to the VLPs is required to elicit protective immunity since it is the major determinant of protection against filovirus infections [32]. The VLP vaccine candidate tested most extensively to date also includes the viral nucleoprotein (NP), which, when included, localizes in the core beneath VP40 [33] and is hypothesized to add the advantage of additional protective T cell epitopes [34]. Vaccination with VLPs has recently been demonstrated to protect nonhuman primates against three different species of ebolaviruses including homologous protection using Ebola virus (EBOV) and Sudan virus (SUDV) VLPs [23,35], as well as heterologous protection against Tai Forest Virus (TAFV) by EBOV VLPs [35]. Vaccination of guinea pigs and cynomolgus macaques with MARV VLPs containing GP, nucleoprotein (NP) and VP40 induces protective immunity against parenteral challenge with both MARV (Musoke and -Ci67 isolates) and RAVV [24]. In the current work, we sought to determine whether MARV VLPs were able to protect against exposure with a lethal dose of aerosolized MARV. This is relevant as aerosolized filoviruses are considered to be potential biothreat agents [36,37].

## 2. Materials and Methods

### 2.1. MARV VLP Production

For a generation of the MARV VLPs, the MARV GP, NP and VP40 genes were inserted into a single baculovirus vector system for expression in insect cells [11,24,38,39]. Sf9 insect cells were infected with the single recombinant baculovirus, and the VLPs were recovered from the culture supernatants by high-speed centrifugation, purified on sucrose gradients, and resuspended in phosphate buffered saline (PBS), as previously described [23,24,38,39,40,41]. Total proteins in the VLP preparations were determined and the VLPs were analyzed by SDS-PAGE/Western blotting and ELISA for filovirus protein content and identity, immunogenicity in mice and endotoxin levels.

### 2.2. Animal Care and Use

Cynomolgus macaques (Macaca fascicularis) were selected as the test species since they are considered the “gold standard” for testing filovirus vaccines. Inclusion Criteria—to be suitable for this study, NHPs were required to be immunocompetent, testable by polyclonal activation of lymphocytes *in vitro* prior to vaccinations, and seronegative for selected retroviruses (simian immunodeficiency virus (SIV), simian retrovirus (SRV) and simian T-cell leukemia virus (STLV)) and filoviruses. Macaques were obtained from Worldwide Primates (Miami, Florida) and were young adult males (>1.5 years) having body weights of >4 kg to <9 kg.

Animals were singly housed in stainless steel cages and supplied with a standard primate diet (Purina 5L07 diet) throughout the study. Water was available *ad libitum*. They were provided toys and treats such as fruit and granola bars. Oral rehydration solution was provided when an animal met the dehydration criteria. Room controls were set to maintain temperatures at 20 to 24 °C (68–75 °F), with a relative humidity of 50% ± 25% with a 12 h light/dark cycle.

Animal care personnel observed the macaques daily for general health, humane treatment, and husbandry conditions. For 21 days after challenge (study days 112–133), animals were also observed at least twice daily by scientific investigators (*i.e.*, health checks were performed a minimum of three times per day). For days 22–28 after challenge (study days 134–140), observations returned to once daily, unless the animal was considered ill. The attending veterinary staff provided routine veterinary medical care during the experiments and were on call 24 hours per day to provide emergency care if required.

Each macaque was anesthetized, weighed, assessed by physical examination, and had its temperature taken and a blood sample collected on study days 0, 14, 28, 42, 56, 70, 84, 105, 112, 115, 117, 119, 122, 126, 133, and 140. Techniques for blood collection and guidance on blood collection volumes were derived from the U.S. Army Medical Research Institute of Infectious Diseases (USAMRIID) Standard Operating Procedures. Blood specimens were collected aseptically from anesthetized nonhuman primates in “Vacutainer” tubes with a 21 gauge 1.5 inch needle via the femoral vein. Blood sample collection was limited to a maximum of 10% of blood volume based on 6.5 mL/kg (e.g., for a 4 kg animal, no more than 26 mL may be collected at once) during a single draw or a total of 10% over a two-week period.

When an animal became clinically ill and met the predetermined criteria for euthanasia, it was first anesthetized and a final blood sample was collected. Subsequently, euthanasia was performed by intravenous or intracardiac administration of a barbiturate overdose.

All macaques that survived until study day 140 (day 28 after MARV challenge) were considered to have survived the viral infection. These animals were then anesthetized, bled, and euthanized as described above.

Partial necropsies were performed on all of the macaques by a board-certified veterinary pathologist. The following tissues from each animal were sampled for histology: axillary lymph node, inguinal lymph node, mandibular lymph node, mesenteric lymph node, liver, spleen, left and right kidney, larynx, thyroid, trachea, lungs, and mediastinal lymph nodes.

This animal research was conducted according to the research protocol approved by the USAMRIID Institutional Animal Care and Use Committee. This was in compliance with the Animal Welfare Act and other federal statutes and regulations relating to animals and experiments involving animals and adhered to the principles stated in the Guide for the Care and Use of Laboratory Animals, National Research Council, 2011. The facility where this research was conducted is fully accredited by the Association for the Assessment and Accreditation of Laboratory Animal Care International. All challenge studies and necropsies were conducted under maximum containment in an animal biosafety level 4 (BSL-4) facility at USAMRIID.

### 2.3. VLP Vaccination

Thirteen macaques received intramuscular injections in the caudal thigh muscle containing 3 mg (total protein) of MARV VLPs and either 0.1 mg of QS-21 adjuvant (Agenus, Inc., Lexington, MA, USA) or 0.5 mg/kg of polyI:C adjuvant (Oncovir, Washington, DC, USA). The three control animals received injections of one of the adjuvants only. Immunizations were performed on study days 0, 42, and 84.

### 2.4. Determination of IgG Antibody Titers against GP and VP40

Pre-challenge blood samples from each macaque were tested by ELISA for serologic responses to filovirus proteins (purified MARV GP amino acid residues 1 to 649 (GPdTM) or VP40) on study days 0, 14, 28, 42, 56, 70, 84 and 105. GPdTM was expressed in 293T cells [42] and purified using a two-step column chromatography method from the supernatants. VP40 was produced and purified as we have previously described [12]. ELISA plates were directly coated with the antigen of interest before application and detection of the antibody samples. The antibodies in unknown samples were tested for binding to the GPdTM or VP40 protein coated on the plates and quantitated based on a standard curve of positive control pooled sera derived from hyperimmune macaques (also referred to as reference detection antibody, RDA). The RDA is well characterized using a 4-parameter (4PL) curve fit and the value of the 4PL curve inflection point was used to establish the number of antibody units for the RDA [13]. Antibody units were then log-transformed and tested using a repeated-measure two-way ANOVA followed by a multiple comparisons test for VP40 where a significant interaction was noted.

### 2.5. Virus Challenge Procedures

MARV was grown on Vero cells (6 total passages) and enumerated using standard plaque assay [43]. Administration of the virus challenge was performed on study day 112 with the challenge material administered in the subcutaneous (SQ) tissues of the left thigh of each animal or via the aerosol route, as previously described [44,45]. The target challenge dose for both SQ and aerosol exposure was 1000 plaque-forming units (pfu)/mL. For the SQ challenge, each macaque received 0.5 mL of challenge stock MARV-Musoke, which upon back-titration revealed an actual challenge dose of 315 pfu/macaque. For aerosol challenge, each macaque was exposed to 10 mL of challenge stock MARV-Musoke using a previously described methodology, and the air in the aerosolization chamber was sampled during each exposure to calculate the actual inhaled dose of virus that each animal received [46,47]. This revealed that the inhaled dose ranged between 40–135 pfu/macaque.

### 2.6. Post-Challenge Sample Analytics

Blood was collected from each animal on the days specified in Section 2.2 above. Clinical chemistry analyses (Complete Metabolic) were performed on serum within 1 hour of blood processing. The following chemical parameters were assessed: sodium (NA), potassium (K), carbon dioxide (CO_2_), bicarbonate (HCO_3_), chloride (CL), glucose (Glu), blood urea nitrogen (BUN), creatinine (Cre), calcium (Ca), albumin (ALB), total protein (TP), alanine aminotransferase (ALT), aspartate aminotransferase (AST), alkaline phosphatase (ALP), and total bilirubin (TBIL). Hematological analysis of whole blood was performed as well and the following parameters were captured: White blood cell count (WBC), red blood cell count (RBC), hemoglobin (Hgb), hematocrit (Hct), mean corpuscular volume (MCV), mean corpuscular hemoglobin (MCH), mean corpuscular hemoglobin concentration (MCHC), platelet count (Plt), percentage of lymphocytes (Ly%) and number of lymphocytes (Ly#). Viremia was determined using standard plaque assay [43].

## 3. Results

### 3.1. Antibody Responses in Vaccinated Macaques

**Figure 1:**
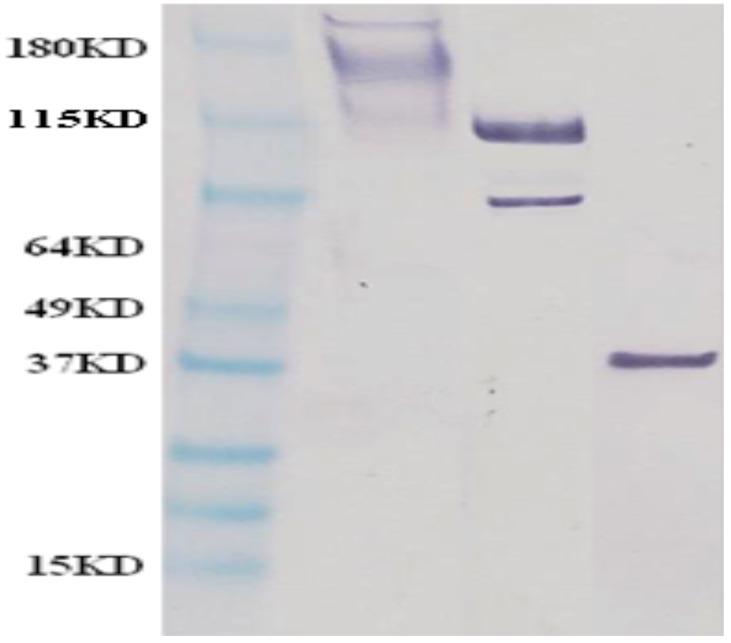
Western blot showing identity of MARV antigens in the MARV VLP preparations used for vaccination. VLP preparations were separated on a SDS-PAGE gel, transferred to nitrocellulose and subjected to immunoblotting using MARV GP—(left sample lane), NP—(middle sample lane) and VP40—(right sample lane) specific antibodies. A molecular mass marker is located in the left most lane.

**Figure 2:**
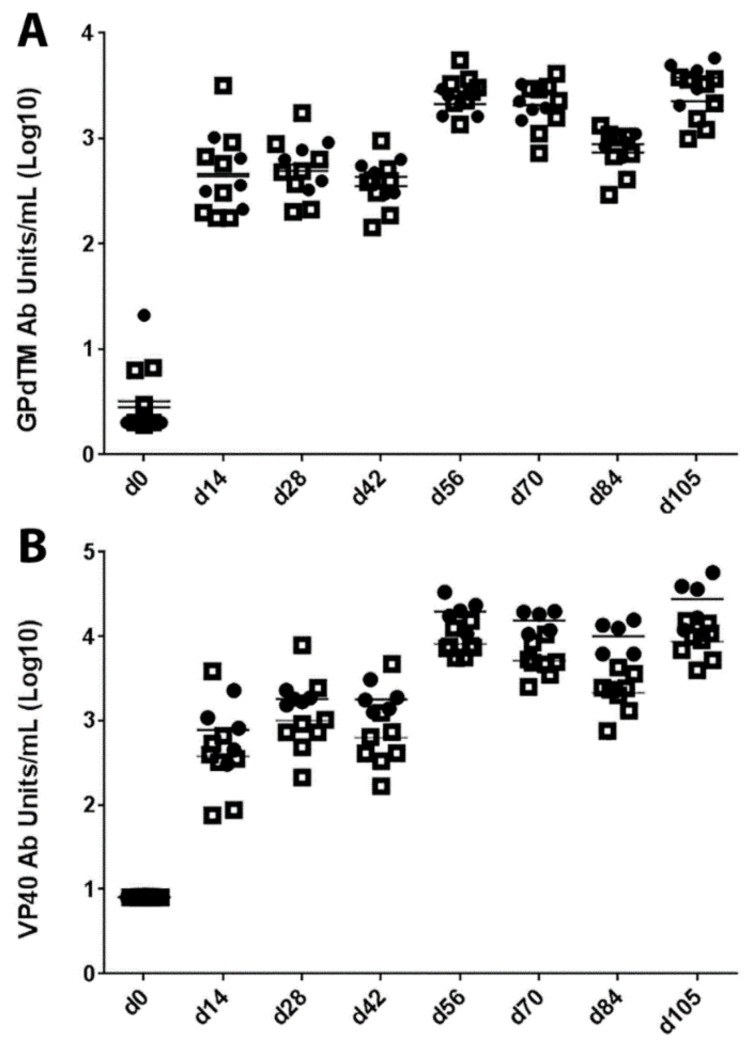
(**A**,**B**) IgG response of nonhuman primates against MARV antigens following MARV VLP vaccination. Serum titers from vaccinated macaques were measured for IgG against purified MARV GPdTM (A) or VP40 (B) by ELISA. The data are expressed as the antibody units for individual animal responses with animals receiving MARV VLP with QS-21 depicted as open squares or those receiving MARV VLPs with polyI:C depicted as closed circles or as the mean antibody units for each group (lines) at each time point the samples were drawn. Vaccination with MARV VLPs combined with either QS-21 or polyI:C exhibited similar responses to the protective GP antigens (*p* = 0.6006) but animals vaccinated with polyI:C had higher responses to the VP40 antigen than those vaccinated with QS-21, specifically at the later time points of days 70, 84 and 105 post vaccination (*p* = 0.0057).

Cynomolgus macaques were vaccinated with MARV VLPs (Figure 1) on study days 0, 42, and 84 and serum antibody titers against purified MARV GP and VP40 were determined for each animal every two to three weeks (study days 0, 14, 28, 42, 56, 70, 84 and 105). In addition to the saponin derivative QS-21, which has been used as an adjuvant for previous filovirus vaccine studies [24], we also examined the ability of polyI:C [48,49,50] as an adjuvant to induce protective immunity of the MARV VLPs. Control animals, which were vaccinated with QS-21 or polyI:C adjuvant alone, did not generate any antibody responses to either MARV GPdTM or VP40 (below the limit of detection at a 1:100 dilution of serum). Results for the VLP-vaccinated animals are presented in Figure 2. The vaccinated animals exhibited similar kinetics of antibody responses to both antigens, with detectable antibody titers at day 14, which waned slightly at day 42 and increased again after the second and third vaccinations were administered on study days 42 and 84 (Figure 2). Animals vaccinated with MARV VLPs with either QS-21 or polyI:C exhibited similar responses to the protective GP antigens (*p* = 0.6006). Animals vaccinated with MARV VLPs and polyI:C had higher responses to the VP40 antigen than those vaccinated with MARV VLP and QS-21, specifically at the later time points of days 70, 84 and 105 post vaccination (*p* = 0.0057).

### 3.2. Quantitation of Virus Inocula

For the SQ challenge, the results of virus back titration indicated that each macaque received 315 pfu of MARV (Table 1).

For aerosol challenge, the results of the back titration indicated that each macaque received between 40 and 135 pfu of MARV (results for each animal are listed in Table 1). The presented dose is calculated for each animal by multiplying the total volume (V_t_) of experimental atmosphere inhaled (V_t_ = V_m_ × length of exposure) by the aerosol concentration (C_e_) (“presented dose”pC_e_ × V_t_). This equation assumes constant minute volume and constant aerosol concentration over time with complete (100%) respiratory deposition. Aerosol concentration is calculated by: (C_sampler_ × V_sampler_)/(Q_sampler_ × t_sampled_); where Cs_ampler_ = the titrated concentration of the sampler, V_sampler_ = the volume of the collection media in the sampler, Q_sampler_ = the flow rate through the sampler, and t_sampled_ = the total time the sample was taken.

The historical average mass median aerodynamic diameter of the generated aerosol particles containing filovirus is approximately 1.4 μm with a geometric standard deviation of 2.1, as measured by a Model 3321 Aerodynamic Particle Sizer (TSI, St. Paul, MN, USA) and by a seven-stage cascade impactor (Intox, Albuquerque, NM, USA).

### 3.3. Post-Challenge Observations

#### 3.3.1. Behavioral and Other Visible Clinical Signs

In Groups 1, 2 and 4, the macaques vaccinated with MARV VLPs and challenged via either aerosol or SQ route, there were no animals with visible clinical signs of filovirus infection. The adjuvant only control macaques presented with typical clinical signs of filovirus infection in macaques. Animal 16-P-S, which was vaccinated with polyI:C alone and challenged by the SQ route exhibited severe depression, moderate rash, and no food intake at day 10 post challenge. Both of the macaques that received QS-21 only (animals 11-Q-A and 15-Q-S) exhibited severe depression, widespread rash, and no food intake at day 10 post challenge. These results are shown in Table 1.

#### 3.3.2. Temperatures

Rectal temperatures of the animals were measured during the challenge phase of the study on days 0, 3, 5, 7, 10, 14, 21 and 28 post challenge. In Group 1 (MARV VLP + Poly-IC adjuvant with aerosol challenge), only one macaque (animal 3-MP-A) recorded a temperature increase of >2 degrees Fahrenheit above baseline measurement. This fever was only present on day 10 post challenge and subsequently returned to normal for the remainder of the study. Body temperatures in the other animals in this group were normal throughout the study. In Group 2 (MARV VLP + QS-21 adjuvant with aerosol challenge), animal 7-MQ-A had a fever on days 10, 14, and 21 post challenge, but its temperature had returned to normal by the end of the study. Body temperatures in the other animals in this group were normal throughout the study. None of the three adjuvant only control animals developed an observed fever. However, animal 16-P-S (polyI:C with SQ challenge) did record a large decrease in temperature of 8 degrees Fahrenheit on day 10 post challenge and was subsequently euthanized.

#### 3.3.3. Hematological and Clinical Chemistry Parameters

Hematology parameters were determined in all surviving animals during the challenge phase of the study on days 0, 3, 5, 7, 10, 14, 21 and 28 post challenge. Abnormal results are summarized in Table 1. In the vaccine Groups 1, 2, and 4, the changes in white blood cell (WBC) counts were unremarkable. For the adjuvant only control groups, all three macaques displayed ≥2× increased WBC counts on study day 10 post infection immediately prior to euthanasia. Platelet levels were also determined. In Group 1, only animal 4-MP-A showed a decrease in platelet count after challenge (day 7 post challenge), which returned to baseline by the next sample date. Other macaques in this group had unremarkable changes in platelet count. In Group 2, animal 10-MQ-A displayed a decrease in platelet count after challenge (day 10 post challenge), which returned to a normal level at the next sampling date. Other macaques in Group 2, as well as in Group 4 and the adjuvant only control groups had unremarkable changes in platelet count. Other than these reported changes in hematology parameters, no additional alterations were noted in any of the groups (a complete list of tests is listed in the methods section).

Serum clinical chemistry parameters were determined on days 0, 3, 5, 7, 10, 14, 21 and 28 post challenge. A complete list of tests is listed in Section 2.6 of the methods. On days 5 and 7 after challenge, animal 3-MP-A in Group 1 (MARV VLP + Poly-IC adjuvant with aerosol challenge) had increased ALT levels above baseline (3× and 2×, respectively) with these levels returning to baseline at the day 10 after challenge time point. There were no other remarkable changes in the clinical chemistry parameters in this animal or in the other macaques in this group. On days 14 and 21 after challenge, animal 6-MQ-A in Group 2 (MARV VLP + QS-21 adjuvant with aerosol challenge) had increased ALP levels (4× and 2×, respectively) with resolution by day 28 after challenge. There were no other remarkable changes in the clinical chemistry parameters in this animal or in the other macaques in this group. The clinical chemistry analyses for the Group 4 monkeys (MARV VLP + QS-21 adjuvant with SQ challenge) did not reveal any abnormalities in any of the animals.

In contrast to the VLP-vaccinated animals, numerous abnormalities in the serum chemistry parameters were detected in each of the macaques that received adjuvant only. On day 7 after viral challenge, all three macaques had increased levels of ALT (2× for all) and AST (5×, 6×, and 7×); increases in these enzymes are indicative of hepatocellular damage. Two of the macaques also had increased ALP levels, which indicate bile stasis, at day 7 post challenge (2× and 4×). On day 10 after challenge and the day of euthanasia, all three of the animals had markedly increased levels of ALT (10×, 21×, and 22×), AST (50×, 60×, and 71×), and ALP (5×, 8×, and 14×). While only one of these macaques had increased levels of CRE at day 7 post challenge (4×), all three animals had increased CRE levels at day 10 post challenge (4×, 6×, and 6×), as well as >2× increase in BUN levels. Increases in CRE and BUN are indicative of compromised kidney function. One animal had decreased levels of glucose at day 7 post challenge (3×), and all three macaques had decreased glucose levels at day 10 post challenge (2×, 5×, and 8×); hypoglycemia often occurs in animals that are anorexic and/or moribund.

#### 3.3.4. Viremia

**Figure 3:**
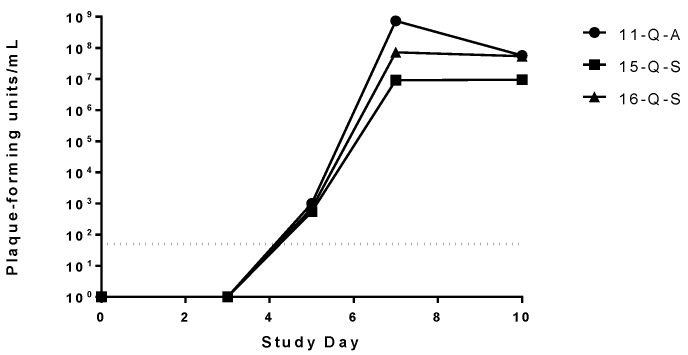
Assessment of viremia in MARV infected macaques. Viral load in serum samples for each animal on various time points after challenge was determined using a standard plaque assay. No virus was detected in any of the MARV VLP vaccinated macaques, regardless of the adjuvant or challenge route used. Viremia of the three control macaques is represented as the values for individual monkeys on each study day after the challenge day. The limit of detection for the plaque assay (50 pfu) is depicted by the dotted line.

Viremia of the challenged macaques was assessed on study days 0, 3, 5, 7, 10, 14, 21 and 28 post challenge. Detectable circulating virus was only present in the three adjuvant only vaccinated animals starting at day 5 post infection, peaking at study day 7 post challenge and leveling out or slightly decreasing on study day 10 post challenge (Figure 3). There was no detectable circulating virus in any of the MARV VLP vaccinated animals at any of the time points tested (data not shown).

#### 3.3.5. Necropsy Findings

**Figure 4:**
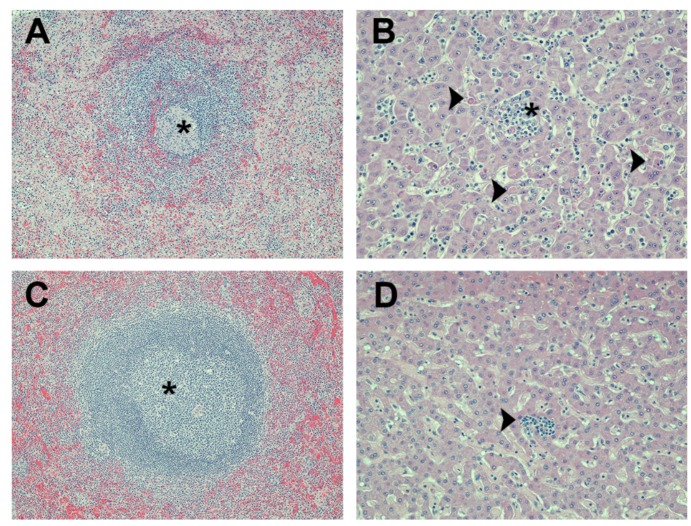
Photomicrographs of liver and spleen from macaques that were challenged with MARV. (**A**) spleen from a control animal (vaccinated with QS-21 adjuvant only and challenged subcutaneously) has a lymphoid nodule with markedly reduced numbers of lymphocytes (*i.e.*, lymphoid depletion) and which is surrounded by pale pink deposits of fibrin in the adjacent red pulp. The center of this nodule (asterisk) has an aggregate of histiocytes and fragments of lysed lymphocytes; (**B**) liver from a control animal (vaccinated with QS-21 adjuvant only and aerosol challenged) has multiple foci of hepatocellular degeneration and necrosis (arrowheads). Numerous neutrophils and monocytes are present in the sinusoidal blood vessels and aggregate around necrotic hepatocytes in one area (asterisk); (**C**) spleen from a VLP-vaccinated animal (Poly I:C adjuvant and aerosol challenged) that survived the viral challenge. A proliferation of lymphocytes (*i.e.*, lymphoid hyperplasia) expands a lymphoid nodule (asterisk); (**D**) liver from a VLP-vaccinated animal (QS-21 adjuvant and aerosol challenged) that survived the viral challenge. Tissue is normal except for a small focus of subacute inflammation (arrowhead). Magnification: 100× for panels (A) and (C); 200× for panels (B) and (D). Hematoxylin and eosin stain was used for tissue sections in all panels.

All three of the adjuvant control monkeys had gross lesions that are typical of those that occur in cynomolgus macaques with lethal MARV infections. These included numerous red cutaneous macules (commonly referred to as a “petechial rash”), a tan to yellow friable liver, an enlarged spleen, and one or more enlarged peripheral lymph nodes. Most of the lesions seen histologically in these animals were also characteristic of MARV infection. Splenic lesions consisted of moderate to marked lymphoid depletion with lymphocyte lysis, acute inflammation, and fibrin deposition (Figure 4A). Varying degrees of lymphoid depletion and lymphocyte lysis were also present in several lymph nodes of these animals. Their livers had multiple foci of hepatocellular degeneration and necrosis accompanied by varying degrees of acute to subacute inflammation (Figure 4B). Marked inflammation and necrosis were present in mediastinal lymph nodes of the aerosol-challenged macaque (animal 11-Q-A); this was not seen in either of the two SQ-challenged animals. The most likely explanation for this is that the mediastinal lymph nodes receive lymphatic drainage from the lungs and that there were high titers of MARV in the pulmonary lymph of the aerosol-challenged animal.

All of the macaques that had been vaccinated with VLPs survived the viral challenge and, as expected, none of these animals had gross lesions suggestive of an active MARV infection at the time of necropsy. The only significant gross lesions seen in these monkeys was mild to moderate enlargement of one or more lymph nodes; this was noted in nine of the 13 animals, which included eight of the aerosol-challenged animals (four monkeys each in Group 1 and Group 2) and one of the SQ-challenged animals (Group 4). Histologically, all 13 monkeys had varying degrees of lymphoid hyperplasia in their spleen (Figure 4C) and lymph nodes. This explains the enlargement of the lymph nodes seen grossly and it is consistent with a reaction to systemic antigenic stimulation associated with vaccination and/or viral challenge. The degree of lymphoid hyperplasia in the mediastinal lymph nodes tended to be greater in the aerosol-challenged animals than in those SQ-challenged, and this was likely associated with high viral titers in the pulmonary lymph following aerosol challenge.

Each of these vaccinated survivors also had one or more foci of very mild subacute inflammation in their liver (Figure 4D). The liver is a target organ of MARV infection, and the lesions present in these monkeys were most likely residual effects of the viral challenge. However, the lesions were too mild to have been of clinical significance and there was no evidence of active viral infection.

Foci of chronic interstitial pneumonia and/or pleuritis were present in seven of the 10 vaccinated survivors that had been aerosol-challenged; four of these were from Group 1 and three were from Group 2. These lesions were probably induced by the aerosol challenge; however, there was no evidence of an active viral infection at the time these animals were euthanized. In five of these seven macaques, the pulmonary lesions were very mild and clinically insignificant. In the other two animals (one from Group 1 and one from Group 2), the pneumonia was more extensive and may have caused clinical signs at some time but it appeared to be resolving and was unlikely to have been clinically significant at the time the animals were euthanized. None of these animals were noted to have clinical signs of respiratory disease during the course of the study; it is also possible that the lung lesions in these monkeys were present before the study began and/or were unrelated to the viral challenge.

There were no other remarkable findings in the survivors.

## 4. Discussion

In this study, cohorts of cynomolgus macaques were vaccinated with VLPs expressing proteins from MARV (Musoke isolate) in the presence of QS-21 or polyI:C adjuvants or were sham-vaccinated with adjuvant alone. Animals were then challenged with the homologous MARV-Musoke strain either by aerosol or SQ route. The outcome indicates that vaccination with MARV VLP with either adjuvant (QS-21 or polyI:C) provides complete protection against challenge with aerosolized MARV-Musoke. Additionally, as expected, MARV VLP with QS-21 adjuvant provided 100% protection against SQ homologous challenge.

This is the first report to date of a subunit vaccine protecting macaques against lethal aerosol infection with MARV. To our knowledge, there is only one other report of a vaccine protecting against aerosolized MARV infection where a multivalent live, replicating vesicular stomatitis virus-based vaccine protected against infection with both Ebola and MARV infections [45]. Additional reports of virus-vectored adenovirus and Venezuelan equine encephalitis virus replicon particle vaccines show protection against multiple ebolaviruses [17,44]. The filovirus VLP platform had not been previously shown to be efficacious against an aerosol infection and further studies to demonstrate protection against other filovirus species such as RAVV, EBOV, SUDV, TAFV and BDBV will be necessary.

In this study, we further broadened the use of novel adjuvants given in conjunction with the filovirus VLPs in nonhuman primates. We had previously shown that Ebola VLPs administered in combination with RIBI or QS-21 adjuvant provide protection of nonhuman primates against homologous and heterologous parenteral infection ([23] and unpublished data). Additionally, MARV VLPs administered in combination with QS-21 protected macaques from parenteral infection with homologous MARV (Musoke or Ci67 strains) or heterologous RAVV [24]. Our recent work showed that addition of polyI:C, R848 or MPL adjuvants increased the potency of Ebola VLPs in mice and guinea pigs [50]. This was the first pilot study using polyI:C with VLP vaccination, and we were encouraged by the protection observed with VLP in the presence of polyI:C when the macaques were challenged by the aerosol route. A cohort of macaques vaccinated with VLP and polyI:C with subsequent infection via SQ route for direct comparisons to our previous study with MARV VLPs and QS-21 was not included in the current studies but should be conducted in the future [24]. The requirement for use of adjuvant for protection by the filovirus VLPs should also be tested.

There are many advantages of the VLP vaccines for filoviruses for future use in humans including manufacturability, lack of ability to replicate, and a known safety profile of this vaccine platform in humans. Their use for protection against multiple species of filoviruses will require at least three components (EBOV, SUDV and MARV VLPs) to broadly protect against the viruses known to be virulent in humans. The inclusion of a component for protection against MARV and RAVV should not be dismissed in future efforts for filovirus vaccines. Disease caused by MARV is just as deadly and spread with the same mechanism as Ebola, and there have been a larger number of MARV outbreaks in the last decade than those caused by EBOV [51]. Fortunately, these recent outbreaks of MARV disease did not affect highly populated areas and the spread could be contained unlike the current EBOV outbreak in West Africa [52,53].

## 5. Conclusions

Vaccination of cynomolgus macaques with MARV VLP with either QS-21 or polyI:C adjuvant provided complete protection against challenge with aerosolized MARV-Musoke.

## Figures and Tables

**Table 1:** Animal group assignment, challenge dose, and outcome of study. Macaques were vaccinated with 3 mg of Marburgvirus-like particles (MARV VLPs) with adjuvant or adjuvant alone three times at 6 week intervals with the viral challenges occurring 4 weeks after the final vaccination. N.S. = no signs; Fever is defined as a temperature more than 2.0 °F over baseline; Temp drop is defined as a temperature less than 5.0 °F below baseline; Moderate rash = areas of red macules covering between 10% and 40% of the skin; Severe rash = areas of red macules covering more than 40% of the skin; ↑, 2- to 3- fold increase; ↑↑, 4- to 5-fold increase; ↑↑↑, >5-fold increase; ↓, 2- to 3- fold decrease; ↓↓↓, >5-fold decrease; Weight loss is shown as percentage compared to weight at study start; BUN, blood urea nitrogen; ALT, alanine aminotransferase; AST, aspartate aminotransferase; ALP, alkaline phosphatase; WBC, white blood cells; Plt, platelet; CRE, creatinine; GLU, glucose.

Group Description	ID	Challenge Route	Challenge Dose (pfu)	Day Post Challenge							Outcome
				0 and 3	5	7	10	14	21	28	
*Group 1: mVLP + Poly-IC adjuvant*	1-MP-A	Aerosol	40	N.S.	N.S.	N.S.	N.S	N.S.	N.S.	N.S.	Survived
	2-MP-A		105	N.S.	N.S.	N.S.	N.S.	N.S.	N.S.	N.S.	Survived
	3-MP-A		122	N.S.	↑ALT	↑ALT	Fever	N.S.	N.S.	N.S.	Survived
	4-MP-A		113	N.S.	N.S.	↓Plt	N.S.	N.S.	N.S.	N.S.	Survived
	5-MP-A		113	N.S.	N.S.	N.S.	N.S.	N.S.	N.S.	N.S.	Survived
*Group 2: mVLP + QS-21 adjuvant*	6-MQ-A	Aerosol	135	N.S.	N.S.	N.S.	N.S.	↑↑ALP	↑ALP	N.S.	Survived
	7-MQ-A		113	N.S.	N.S.	N.S.	Fever	Fever	Fever	N.S.	Survived
	8-MQ-A		83	N.S.	N.S.	N.S.	N.S.	N.S.	N.S	N.S.	Survived
	9-MQ-A		93	9.5% weight loss (day 3)	N.S.	N.S.	N.S.	N.S.	N.S.	N.S.	Survived
	10-MQ-A		117	N.S.	N.S.	N.S.	↑WBC, ↓Plt	N.S.	N.S.	N.S.	Survived
*Group 3: QS-21 only*	11-Q-A	Aerosol	113	N.S.	N.S.	↑↑ALP, ↑ALT, ↑↑↑AST	Severely depressed, severe rash, no food, ↑↑WBC, ↑↑↑BUN, ↑↑↑ALP, ↑↑↑ALT, ↑↑↑AST, ↑↑CRE, ↓GLU				Euthanized day 122
*Group 4: mVLP + QS-21 adjuvant*	12-MQ-S	SQ	315	N.S.	N.S.	N.S.	N.S.	N.S.	N.S.	N.S.	Survived
	13-MQ-S		315	N.S.	N.S.	N.S.	N.S.	N.S.	N.S.	N.S.	Survived
	14-MQ-S		315	N.S.	N.S.	N.S.	N.S.	N.S.	N.S.	N.S.	Survived
*Group 5: QS-21 only*	15-Q-S	SQ	315	N.S.	N.S.	↑ALP, ↑ALT, ↑↑↑AST, ↑↑CRE, ↓GLU	Severely depressed, severe rash, no food, ↑WBC, ↑↑BUN, ↑↑↑ALP, ↑↑↑ALT, ↑↑↑AST, ↑↑↑CRE, ↓↓↓GLU				Euthanized day 122
*Group 6: Poly-IC only*	16-P-S	SQ	315	N.S.	N.S.	↑ALT, ↑↑↑AST	Severely depressed, moderate rash, no food, ↑WBC, ↑BUN, ↑↑↑ALP, ↑↑↑ALT, ↑↑↑AST, ↑↑↑CRE, ↓↓↓GLU, Temp Drop				Euthanized day 122

## References

[1] Brauburger K., Hume A.J., Muhlberger E., Olejnik J. (2012). Forty-five years of marburg virus research. Viruses.

[2] Martini G.A., Siegert R. (1971). Marburg Virus Disease.

[3] Smith D.H., Johnson B.K., Isaacson M., Swanapoel R., Johnson K.M., Killey M., Bagshawe A., Siongok T., Keruga W.K. (1982). Marburg-virus disease in Kenya. Lancet.

[4] Kuhn J.H., Becker S., Ebihara H., Geisbert T.W., Johnson K.M., Kawaoka Y., Lipkin W.I., Negredo A.I., Netesov S.V., Nichol S.T. (2010). Proposal for a revised taxonomy of the family filoviridae: Classification, names of taxa and viruses, and virus abbreviations. Arch. Virol..

[5] Zeller H. (2000). Lessons from the marburg virus epidemic in Durba, Democratic Republic of the Congo (1998–2000). Med. Trop. (Mars).

[6] Colebunders R., Sleurs H., Pirard P., Borchert M., Libande M., Mustin J.P., Tshomba A., Kinuani L., Olinda L.A., Tshioko F. (2004). Organisation of health care during an outbreak of Marburg haemorrhagic fever in the Democratic Republic of Congo, 1999. J. Infect..

[7] Towner J.S., Khristova M.L., Sealy T.K., Vincent M.J., Erickson B.R., Bawiec D.A., Hartman A.L., Comer J.A., Zaki S.R., Stroher U. (2006). Marburgvirus genomics and association with a large hemorrhagic fever outbreak in Angola. J. Virol..

[8] Mbonye A., Wamala J., Winyi K., Tugumizemo V., Aceng J., Makumbi I. (2012). Repeated outbreaks of viral hemorrhagic fevers in Uganda. Afr. health sci..

[9] Geisbert T.W., Bausch D.G., Feldmann H. (2010). Prospects for immunisation against Marburg and Ebola viruses. Rev. Med. Virol..

[10] Warfield K.L., Jaax N.K., Deal E.M., Swenson D.L., Larsen T., Bavari S., Swearengen J.R. (2005). Viral hemorrhagic fevers. Biodefense: Research Methodology and Animal Models.

[11] Bradfute S.B., Dye J.M., Bavari S. (2011). Filovirus vaccines. Hum. Vaccines.

[12] Geisbert T.W., Pushko P., Anderson K., Smith J., Davis K.J., Jahrling P.B. (2002). Evaluation in nonhuman primates of vaccines against Ebola virus. Emerg. Infect. Dis..

[13] Hevey M., Negley D., Vanderzanden L., Tammariello R.F., Geisbert J., Schmaljohn C., Smith J.F., Jahrling P.B., Schmaljohn A. (2002). Marburg virus vaccines, comparing classical and new approaches. Vaccine.

[14] Cooper C.L., Bavari S. (2015). A race for an Ebola vaccine: Promises and obstacles. Trends Microbiol..

[15] Hevey M., Negley D., Pushko P., Smith J., Schmaljohn A. (1998). Marburg virus vaccines based upon alphavirus replicons protect guinea pigs and nonhuman primates. Virology.

[16] Sullivan N.J., Sanchez A., Rollin P.E., Yang Z.Y., Nabel G.J. (2000). Development of a preventive vaccine for Ebola virus infection in primates. Nature.

[17] Herbert A.S., Kuehne A.I., Barth J.F., Ortiz R.A., Nichols D.K., Zak S.E., Stonier S.W., Muhammad M.A., Bakken R.R., Prugar L.I. (2013). Venezuelan equine encephalitis virus replicon particle vaccine protects nonhuman primates from intramuscular and aerosol challenge with ebolavirus. J. Virol..

[18] Geisbert T.W., Bailey M., Geisbert J.B., Asiedu C., Roederer M., Grazia-Pau M., Custers J., Jahrling P., Goudsmit J., Koup R. (2010). Vector choice determines immunogenicity and potency of genetic vaccines against Angola Marburg virus in nonhuman primates. J. Virol..

[19] Jones S.M., Feldmann H., Stroher U., Geisbert J.B., Fernando L., Grolla A., Klenk H.D., Sullivan N.J., Volchkov V.E., Fritz E.A. (2005). Live attenuated recombinant vaccine protects nonhuman primates against Ebola and Marburg viruses. Nat. Med..

[20] Geisbert T.W., Geisbert J.B., Leung A., Daddario-DiCaprio K.M., Hensley L.E., Grolla A., Feldmann H. (2009). Single-injection vaccine protects nonhuman primates against infection with Marburg virus and three species of Ebola virus. J. Virol..

[21] Bukreyev A., Rollin P.E., Tate M.K., Yang L., Zaki S.R., Shieh W.J., Murphy B.R., Collins P.L., Sanchez A. (2007). Successful topical respiratory tract immunization of primates against Ebola virus. J. Virol..

[22] Blaney J.E., Marzi A., Willet M., Papaneri A.B., Wirblich C., Feldmann F., Holbrook M., Jahrling P., Feldmann H., Schnell M.J. (2013). Antibody quality and protection from lethal Ebola virus challenge in nonhuman primates immunized with rabies virus based bivalent vaccine. PLoS Pathog..

[23] Warfield K.L., Swenson D.L., Olinger G.G., Kalina W.V., Aman M.J., Bavari S. (2007). Ebola virus-like particle-based vaccine protects nonhuman primates against lethal ebola virus challenge. J. Infect. Dis..

[24] Swenson D.L., Warfield K.L., Larsen T., Alves D.A., Coberley S.S., Bavari S. (2008). Monovalent virus-like particle vaccine protects guinea pigs and nonhuman primates against infection with multiple Marburg viruses. Expert Rev. Vaccines.

[25] Buonaguro L., Tornesello M.L., Buonaguro F.M. (2010). Virus-like particles as particulate vaccines. Curr. HIV Res..

[26] Noad R., Roy P. (2003). Virus-like particles as immunogens. Trends Microbiol..

[27] Naslund J., Lagerqvist N., Habjan M., Lundkvist A., Evander M., Ahlm C., Weber F., Bucht G. (2009). Vaccination with virus-like particles protects mice from lethal infection of rift valley fever virus. Virology.

[28] Conner M.E., Zarley C.D., Hu B., Parsons S., Drabinski D., Greiner S., Smith R., Jiang B., Corsaro B., Barniak V. (1996). Virus-like particles as a rotavirus subunit vaccine. J. Infect Dis..

[29] Tacket C.O., Sztein M.B., Losonsky G.A., Wasserman S.S., Estes M.K. (2003). Humoral, mucosal, and cellular immune responses to oral norwalk virus-like particles in volunteers. Clin. Immunol..

[30] Roldao A., Mellado M.C., Castilho L.R., Carrondo M.J., Alves P.M. (2010). Virus-like particles in vaccine development. Expert Rev. Vaccines.

[31] Bavari S., Bosio C.M., Wiegand E., Ruthel G., Will A.B., Geisbert T.W., Hevey M., Schmaljohn C., Schmaljohn A., Aman M.J. (2002). Lipid raft microdomains: A gateway for compartmentalized trafficking of Ebola and Marburg viruses. J. Exp. Med..

[32] Swenson D.L., Warfield K.L., Negley D.L., Schmaljohn A., Aman M.J., Bavari S. (2005). Virus-like particles exhibit potential as a pan-filovirus vaccine for both ebola and Marburg viral infections. Vaccine.

[33] Noda T., Watanabe S., Sagara H., Kawaoka Y. (2007). Mapping of the VP40-binding regions of the nucleoprotein of Ebola virus. J. Virol..

[34] Wilson J.A., Hart M.K. (2001). Protection from ebola virus mediated by cytotoxic t lymphocytes specific for the viral nucleoprotein. J. Virol..

[35] Warfield K.L., Dye J.M., Wells J.B., Unfer R.C., Holtsberg F.W., Shulenin S., Vu H., Swenson D.L., Bavari S., Aman M.J. (2014). Homologous and heterologous protection of nonhuman primates by Zaire and Sudan ebolavirus virus-like particles. PLoS ONE.

[36] Jaax N.K., Davis K.J., Geisbert T.J., Vogel P., Jaax G.P., Topper M., Jahrling P.B. (1996). Lethal experimental infection of rhesus monkeys with ebola-Zaire (mayinga) virus by the oral and conjunctival route of exposure. Arch. Pathol. Lab. Med..

[37] Leffel E.K., Reed D.S. (2004). Marburg and Ebola viruses as aerosol threats. Biosecur. Bioterror..

[38] Warfield K.L., Swenson D.L., Negley D.L., Schmaljohn A.L., Aman M.J., Bavari S. (2004). Marburg virus-like particles protect guinea pigs from lethal marburg virus infection. Vaccine.

[39] Swenson D.L., Warfield K.L., Kuehl K., Larsen T., Hevey M.C., Schmaljohn A., Bavari S., Aman M.J. (2004). Generation of Marburg virus-like particles by co-expression of glycoprotein and matrix protein. FEMS Immunol. Med. Microbiol..

[40] Warfield K.L., Bosio C.M., Welcher B.C., Deal E.M., Mohamadzadeh M., Schmaljohn A., Aman M.J., Bavari S. (2003). Ebola virus-like particles protect from lethal Ebola virus infection. Proc. Natl. Acad. Sci..

[41] Warfield K.L., Posten N.A., Swenson D.L., Olinger G.G., Esposito D., Gillette W.K., Hopkins R.F., Costantino J., Panchal R.G., Hartley J.L. (2007). Filovirus-like particles produced in insect cells: Immunogenicity and protection in rodents. J. Infect. Dis..

[42] Lee J.E., Fusco M.L., Hessell A.J., Oswald W.B., Burton D.R., Saphire E.O. (2008). Structure of the ebola virus glycoprotein bound to an antibody from a human survivor. Nature.

[43] Moe J.B., Lambert R.D., Lupton H.W. (1981). Plaque assay for Ebola virus. J. Clin. Microbiol..

[44] Pratt W.D., Wang D., Nichols D.K., Luo M., Woraratanadharm J., Dye J.M., Holman D.H., Dong J.Y. (2010). Protection of nonhuman primates against two species of Ebola virus infection with a single complex adenovirus vector. Clin. Vaccine Immunol..

[45] Geisbert T.W., Daddario-Dicaprio K.M., Geisbert J.B., Reed D.S., Feldmann F., Grolla A., Stroher U., Fritz E.A., Hensley L.E., Jones S.M. (2008). Vesicular stomatitis virus-based vaccines protect nonhuman primates against aerosol challenge with Ebola and Marburg viruses. Vaccine.

[46] Hartings J.M., Roy C.J. (2004). The automated bioaerosol exposure system: Preclinical platform development and a respiratory dosimetry application with nonhuman primates. J. Pharmacol. Toxicol. Methods.

[47] Roy C.J., Pitt L.M., Swearengen J.R. (2006). Infectious disease aerobiology: Aerosol challenge methods. Biodefense: Research Methodology and Animal Models.

[48] Stahl-Hennig C., Eisenblatter M., Jasny E., Rzehak T., Tenner-Racz K., Trumpfheller C., Salazar A.M., Uberla K., Nieto K., Kleinschmidt J. (2009). Synthetic double-stranded rnas are adjuvants for the induction of t helper 1 and humoral immune responses to human Papillomavirus in rhesus macaques. PLoS Pathog..

[49] Longhi M.P., Trumpfheller C., Idoyaga J., Caskey M., Matos I., Kluger C., Salazar A.M., Colonna M., Steinman R.M. (2009). Dendritic cells require a systemic type I interferon response to mature and induce CD4+ TH1 immunity with poly ic as adjuvant. J. Exp. Med..

[50] Martins K.A., Steffens J.T., van Tongeren S.A., Wells J.B., Bergeron A.A., Dickson S.P., Dye J.M., Salazar A.M., Bavari S. (2014). Toll-like receptor agonist augments virus-like particle-mediated protection from Ebola virus with transient immune activation. PLoS ONE.

[51] Kuhn J.H., Dodd L.E., Wahl-Jensen V., Radoshitzky S.R., Bavari S., Jahrling P.B. (2011). Evaluation of perceived threat differences posed by filovirus variants. Biosecur. Bioterror..

[52] Stephenson J. (2014). Largest-ever Ebola outbreak still simmering in West Africa. JAMA.

[53] Gire S.K., Goba A., Andersen K.G., Sealfon R.S., Park D.J., Kanneh L., Jalloh S., Momoh M., Fullah M., Dudas G. (2014). Genomic surveillance elucidates Ebola virus origin and transmission during the 2014 outbreak. Science.

